# Association of Cooking Patterns with Inflammatory and Cardio-Metabolic Risk Biomarkers

**DOI:** 10.3390/nu13020633

**Published:** 2021-02-16

**Authors:** Belén Moreno-Franco, Montserrat Rodríguez-Ayala, Carolina Donat-Vargas, Helena Sandoval-Insausti, Jimena Rey-García, Esther Lopez-Garcia, José R. Banegas, Fernando Rodríguez-Artalejo, Pilar Guallar-Castillón

**Affiliations:** 1 Department of Microbiology, Pediatrics, Radiology and Public Health, Universidad de Zaragoza, 50009 Zaragoza, Spain; mbmoreno@unizar.es; 2 Instituto de Investigación Sanitaria Aragón, Hospital Universitario Miguel Servet, 50009 Zaragoza, Spain; 3 Department of Preventive Medicine and Public Health, School of Medicine, Universidad Autónoma de Madrid-IdiPAZ and CIBERESP (CIBER of Epidemiology and Public Health), 28029 Madrid, Spain; ms.rodriguezayala@gmail.com (M.R.-A.); carolina.donat@imdea.org (C.D.-V.); helenagabar@gmail.com (H.S.-I.); jimena.reygarcia@gmail.com (J.R.-G.); esther.lopez@uam.es (E.L.-G.); joseramon.banegas@uam.es (J.R.B.); fernando.artalejo@uam.es (F.R.-A.); 4 IMDEA-Food Institute, CEI UAM+CSIC, 28049 Madrid, Spain; 5 Unit of Nutritional Epidemiology, Institute of Environmental Medicine, Karolinska Institutet, 17177 Stockholm, Sweden; 6 Department of Nutrition, Harvard T.H. Chan School of Public Health, Boston, MA 02115, USA; 7 Internal Medicine Department, Ramón y Cajal University Hospital, 28034 Madrid, Spain

**Keywords:** cardiovascular risk factors, inflammatory markers, dietary patterns, cooking methods, food preservation

## Abstract

Diet has been clearly associated with cardiovascular disease, but few studies focus on the influence of cooking and food preservation methods on health. The aim of this study was to describe cooking and food preservation patterns, as well as to examine their association with inflammatory and cardio-metabolic biomarkers in the Spanish adult population. A cross-sectional study of 10,010 individuals, representative of the Spanish population, aged 18 years or over was performed using data from the ENRICA study. Food consumption data were collected through a face-to-face dietary history. Cooking and food preservation patterns were identified by factor analysis with varimax rotation. Linear regression models adjusted for main confounders were built. Four cooking and food preservation patterns were identified. The Spanish traditional pattern (positively correlated with boiling and sautéing, brining, and light frying) tends to be cardio-metabolically beneficial (with a reduction in C-reactive protein (−7.69%)), except for high density lipoprotein cholesterol (HDL-c), insulin levels, and anthropometrics. The health-conscious pattern (negatively correlated with battering, frying, and stewing) tends to improve renal function (with a reduction in urine albumin (−9.60%) and the urine albumin/creatinine ratio (−4.82%)). The youth-style pattern (positively correlated with soft drinks and distilled alcoholic drinks and negatively with raw food consumption) tends to be associated with good cardio-metabolic health except, for lower HDL-c (−6.12%), higher insulin (+6.35%), and higher urine albumin (+27.8%) levels. The social business pattern (positively correlated with the consumption of fermented alcoholic drinks, food cured with salt or smoke, and cured cheese) tends to be detrimental for the lipid profile (except HDL-c), renal function (urine albumin +8.04%), diastolic blood pressure (+2.48%), and anthropometrics. Cooking and food preservation patterns showed a relationship with inflammatory and cardio-metabolic health biomarkers. The Spanish traditional pattern and the health-conscious pattern were associated with beneficial effects on health and should be promoted. The youth-style pattern calls attention to some concerns, and the social business pattern was the most detrimental one. These findings support the influence of cooking and preservation patterns on health.

## 1. Introduction

Unhealthy eating is associated with major causes of death, such as cardiovascular disease (CVD) and some types of cancer [1]. Food consumption guidelines for chronic disease prevention base their recommendations on data exclusively focused on nutrients, foods, food groups, and, more recently, dietary patterns; however, they hardly take into account the influence of cooking and food preservation methods on health. 

Cooking and food preservation methods modify the organoleptic conditions of food, making it more palatable, while influencing the bioavailability of nutrients, vitamins, and minerals. At the same time, some cooking and food preservation methods, (as in frying) increase fat and energy content, as well as the possible presence of toxic elements that may result, for example, from the cooking and reheating of food [2]. 

Frying is the cooking method most widely studied. When a food is fried, water is replaced by fat, and the composition of the food is modified. There is also an increase in trans-fatty acids as a result of deep frying [3]. The first relevant article on the health effects of frying was a cross-sectional analysis of 30,000 middle-aged adults. An increase in overall obesity and central obesity was observed among those with a high intake of fried food [4]. However, in longitudinal analyses, fried food consumption was not associated with coronary heart disease, stroke, or all-cause mortality [5,6]. Moreover, in a prospective cohort, a high consumption of fried food was also linked with weight gain, central obesity, and hypertension [7,8,9]. Other longitudinal analyses [10,11] have shown an association of fried food consumption with diabetes, CVD, and heart failure [12]. Thus, based on this evidence, the current recommendation is to consume fried food in moderation, while emphasizing an overall healthy diet [13].

Other cooking methods have barely been studied. Boiling and other cooking methods that use the application of heat lead to degradation of vitamins, as well as an increase in the glycemic index, leading to faster absorption of sugar [14], as well as the formation of toxic substances such as acrylamide when heating foods rich in starch [15] or heterocyclic amines when applying heat to meat products [16]. Furthermore, the increase in salt intake resulting from preservation methods such as brining, salt curing, or canning [17] are clear examples of how industrial processing has an impact on health. In addition, a result of this industrial processing is an increase in oxidative stress and inflammation [18]. On the contrary, diets composed exclusively of raw food have shown an association with negative health outcomes [19,20]. Research on other forms of cooking is very limited and mainly focuses on specific foods [21,22,23,24,25].

The study of dietary patterns provides a global approach concerning the association between food consumption and chronic diseases. Therefore, it seems reasonable to include the influence of cooking and food preservation methods when assessing diet as a whole. The aim of this study is to identify cooking and food preservation patterns in the Spanish adult population and to examine their association with inflammatory and cardio-metabolic biomarkers. Cooking methods, beyond the consumption of fried foods, are little analyzed and have never been studied as a pattern.

## 2. Materials and Methods

### 2.1. Study Design, Setting, and Participants

Data were taken from the ENRICA study, whose methods have been described elsewhere [26]. In brief, this cohort was set up in 2008–2010 with 12,948 individuals aged ≥18 years, representative of the Spanish population. The ENRICA study was approved by the Clinical Research Ethics Committee of La Paz University Hospital (ethics approval code HULP-PI-1793). Study participants gave informed written consent.

### 2.2. Dietary Assessment

Food consumption data were collected with a validated face-to-face computerized diet history [27] obtained by trained interviewers. The dietary history allowed for the collection of standardized data on 860 foods. Each food was assigned a cooking or preservation method, including mixed forms of cooking (for example, vegetables that are first boiled and then sautéed) (Table A1). The software includes 127 sets of digitized photographs to help quantify the size of food portions. Habitual food consumption takes into account a typical week in the preceding year, and every food consumed at least once every 15 days was considered. The intake of macronutrients, micronutrients, and minerals from the foods was estimated using Spanish composition tables [28].

Twenty-four possible cooking and food preservation methods were assessed: fifteen cooking methods (raw (not cooked), dairy products (including milk, yogurts, and fresh uncured cheese), boiling, roasting, pan-frying, frying, toasting, sautéing, stewing, boiling and then sautéing, light frying (*sofrito*), battering (bread crumbed), steaming, barbecuing, and microwaving), five preserving methods (curing cheese, curing with salt or smoke, canning in oil, canning not in oil, and brining), and four groups for beverages (soft drinks, fermented alcoholic drinks, coffee or tea, and distilled alcoholic drinks).

### 2.3. Anthropometric, Clinical, and Biochemical Characteristics

Height was measured twice with portable extendable stadiometers (model Ka We 44 444Seca), weight with an electronic scale (precision 100 g), and waist and hip circumferences with a flexible inelastic belt-type tape, following standardized protocols [29]. The weight of participants was taken in the morning, after 12 h of fasting, and in light clothes. The body mass index (BMI) was calculated as weight in kilograms divided by height in meters-squared. Blood pressure was measured using standard procedures [30] and validated automatic devices (Omron M6) with appropriate cuffs of three sizes. 

Biomarkers of inflammation and cardio-metabolic risk were determined in 12 h fasting blood samples. High-sensitivity C-reactive protein (Hs-CRP) was measured by latex-enhanced nephelometry and fibrinogen by the coagulation method. Total cholesterol was measured by enzymatic methods using cholesterol esterase and cholesterol oxidase; high density lipoprotein cholesterol (HDL-c) by the direct method by elimination/catalase, and triglycerides by the glycerol phosphate oxidase method. Low density lipoprotein cholesterol (LDL-c) levels were calculated with the Friedewald equation [31]. Fasting glucose was measured by the glucose oxidase method and insulin by immunoradiometric assay. Homeostatic model assessment for insulin resistance (HOMA-IR) was calculated by multiplying fasting serum insulin (μIU/mL) by fasting serum glucose (mg/dL) and dividing by 405 [32]. Uric acid was measured by the uricase-peroxidase technique, creatinine by the Jaffé alkaline picrate kinetic reaction, and albumin in urine by polyethylene glycol (PEG)-enhanced immunoturbidimetry. 

All the laboratory determinations were performed centrally at the Center of Biological Diagnosis of the Clinic Hospital in Barcelona according to standard procedures and using appropriate quality controls.

### 2.4. Other Variables

Participants reported their sex, age, educational level (primary, secondary, and university), and smoking status (current, former, or never smoker). Physical activity at leisure time was assessed with the EPIC (European Prospective Investigation into Cancer and Nutrition) Spain cohort questionnaire [33] and expressed in metabolic equivalents (METs)-hours/week [34]. The number of hours per week spent watching TV was also reported. The interviewer collected the following self-reported diseases diagnosed by a physician: pneumonia, asthma or chronic bronchitis, heart infarction, stroke or heart failure, osteoarthritis/arthritis, hip fracture, cholecystolithiasis, intestinal polyps, stomach or duodenal ulcer, cirrhosis, urinary tract infection, cataract, depression requiring drug treatment, Parkinson’s disease, dementia, Alzheimer’s disease, and periodontal disease.

### 2.5. Statistical Analysis 

The study sample comprised 12,948 individuals. Of these, those with a lack of data on diet, biomarkers of inflammation and cardio-metabolic risk, or other variables were excluded. Thus, the final analytical sample included 10,010 individuals.

An exploratory factor analysis with varimax rotation was carried out to identify cooking and food preservation patterns. The factors were rotated by the varimax function. An orthogonal rotation was made to assimilate each variable with an axis, reducing the complexity of the patterns and facilitating their interpretation [35]. Positive loading factors indicated that the individual cooking or preservation method was directly associated with the factors, while negative loading factors indicated an inverse association with the factors. Factorial loadings above 0.3 were considered to be major contributors to the factors [36]. Cooking patterns were named according to the cooking and food preservation methods that contributed the most. The associations between each cooking and preservation pattern with inflammatory and cardio-metabolic risk biomarkers were evaluated by multivariate linear regression. 

Each participant received a score for each pattern that indicated the degree of adherence of that participant to that specific pattern. So, cooking and preservation patterns were modeled in sex-specific quartiles, and the lowest quartile was used as reference. Inflammatory and cardio-metabolic biomarkers that did not follow a normal distribution were log-transformed prior to statistical analysis. For each quartile, the adjusted marginals of the biomarkers were calculated. The geometric mean (±SD) was obtained by inverse transformation. As a summary to aid interpretation, the relative differences between extreme quartiles were calculated and presented in a summary table ((value in the first quartile-value in the last quartile)/value in the first quartiles). The linear trend was calculated considering quartiles as a continuous variable.

The linear regression models were adjusted for sex, age, educational level (primary, secondary, or university), smoking status (current, former, or never smoker), physical activity (MET-h/week), time spent watching TV (h/week), energy intake (kcal/day), and morbidity (to have at least one of the considered comorbidities). Only relative changes among extreme quartiles greater than 1% were considered for the description of the trends. All analyses were performed with STATA (version 15.0; StataCorp). Statistical tests were 2-tailed with a significance level of 5%.

## 3. Results

The exploratory factorial analysis identified four cooking and food preservation patterns. The first pattern was positively associated with sautéing and boiling, brining, light frying (*sofrito*), roasting, boiling, canning in oil, and frying and negatively associated with pan-frying, and this was named the Spanish traditional pattern. The second factor was positively associated with toasting, brining, steaming, and canning in oil and negatively associated with battering, frying, and stewing, and this was named the health-conscious pattern. The third factor was positively associated with soft drinks and distilled alcoholic drinks and negatively associated with the consumption of raw food, coffee, and tea, and this was named the youth-style pattern. The fourth factor was positively associated with fermented alcoholic drinks, curing (salted or smoked), curing cheese, and distilled alcoholic drinks and negatively associated with the intake of dairy products, and this was named the social business pattern (Table 1). These four patterns accounted for 22% of the variance of total cooking and food preservation methods.

Participants with a greater adherence to the Spanish traditional pattern were less physically active, watched less TV, and reported a greater intake of energy than those with a lower adherence to this pattern. Participants with a greater adherence to the health-conscious pattern were more educated, less frequently smoked, were more physically active, watched less TV, and reported a lower energy intake. Participants with a greater adherence to the youth-style pattern were younger, more educated, and more frequent smokers and performed more physical activity, watched less TV, reported a higher energy intake, and had fewer comorbidities. Finally, participants with a greater adherence to the social business pattern were more educated, more frequently smoked, performed less physical activity, spent less time watching TV, had a higher energy intake, and had fewer comorbidities compared to those with a lower adherence to this pattern (Table 2).

Regarding the association between the different identified patterns and the measured biomarkers, participants with a higher adherence to the Spanish traditional pattern had lower levels of Hs-CRP (−7.69%), fibrinogen (−1.79%), total cholesterol (−1.39%), LDL-c (−1.44%), glucose (−1.09%), and urine albumin/creatinine ratio (−1.87%). On the contrary, they tended to present a detrimental profile for HDL-c (−2.28%), insulin (+1.58%), and anthropometric measurements (BMI +0.97%; waist circumference +1.75%; waist-to-hip ratio +1.13%) (Table 3). The health-conscious pattern was associated with lower levels of urine albumin (−9.60%) and a low urine albumin/creatinine ratio (−4.82%) (Table 4). The youth-style pattern was associated with lower levels of Hs-CRP (−7.69%), fibrinogen (−5.62%), total cholesterol (−9.09%), LDL-c (−11.7%), triglycerides (−5.03%), glucose (−6.72%), uric acid (−4.42%), urine albumin/creatinine ratio (−6.36%), systolic and diastolic blood pressure (−5.82% and −4.37% respectively), BMI (−4.26%), waist circumference (−5.19%), and waist-to-hip ratio (−4.49%). In contrast, it showed lower levels of HDL-c (−6.12%), insulin (+6.35%), and urine albumin (+27.8%) (Table 5). The social business pattern was associated with lower levels of fibrinogen (−3.56%), insulin (5.81%), and HOMA-IR (−5.71%). On the contrary, it presented higher levels of total cholesterol, LDL-c (+3.94%), HDL-c (+3.51%), triglycerides (+3.68%), uric acid (+4.42%), urine albumin (+8.04), diastolic blood pressure (+2.48%), and anthropometrics (BMI +1.44%; waist circumference +1.06%; waist-to-hip ratio +1.13%) (Table 6 and Table A2).

## 4. Discussion

Four different cooking and food preservation patterns were identified. The derived patterns did not share almost any cooking or preservation method and were linked to different types of social behaviors. The Spanish traditional pattern tended to be beneficial, except for HDL-c, insulin, and anthropometrics. The health-conscious pattern showed better renal function. The youth-style pattern tended to be associated with beneficial cardio-metabolic health except for a decrease in HDL-c and an increase in insulin and urine albumin levels. Finally, the social business pattern tended to associate unfavorably with the lipid profile (except HDL-c), uric acid, urine albumin, diastolic blood pressure, and anthropometrics; on the contrary, it also tended to correlate with lower fibrinogen levels.

The cooking methods of the Spanish traditional pattern correspond with the traditional Spanish and Mediterranean cuisine, which typically includes sautéing and boiling, brining, light frying (*sofrito*), and frying. In line with our results, numerous studies have shown that adherence to a Mediterranean dietary pattern, characterized by a high consumption of fresh and not processed foods mostly of plant origin, has been associated with health benefits, including clear anti-inflammatory and heart-healthy properties [37]. This Spanish traditional pattern was also associated with a detrimental anthropometric profile as well as high insulin resistance that could be explained by the high prevalence of obesity and metabolic syndrome in Spain [29]. Another possible explanation for these associations may be due to a higher energy intake associated with an elaborate and palatable diet made at home, including high-density food from frying and the consumption of food canned in oil, not accompanied by sufficient physical activity. In addition, there is some evidence that a greater intake of olive oil (as in the Mediterranean diet) is associated with obesity [7]. Additionally, those with a higher adherence to this pattern presented a decrease in HDL-c that could be partially explained by lower physical activity and low alcohol consumption, even when the models were adjusted for these factors. It is worth noting that brined foods, such as vegetables [38], are rich in nitrates and nitrites, which are related to cardiovascular benefits [39]. In addition, dietary phenolic compounds present in *sofrito* enhance glucose metabolism [40]. When considered as a whole, this pattern was healthy (except for anthropometrics).

The health-conscious pattern included simpler food elaboration levels (toasting or steaming with no added oil), avoiding cooking methods such as battering, frying, or stewing. This pattern tended to improve renal function (low urine albumin and albumin/creatinine ratio). This could be explained by the fact that less elaborated food (toasted, brined, steamed, food canned in oil) leads to better preservation of antioxidant and mineral compounds (i.e. those present in vegetable fiber) that are associated with vascular benefits [41]. Moreover, this pattern was inversely related to energy intake, being the only pattern with this characteristic. A more active lifestyle and other healthy habits among these participants, such as not smoking, could also partially explain the beneficial effects of this pattern.

The youth-style pattern with higher adherence among younger participants (mean age 55 and 36 years old in Q1 and Q4, respectively) was strongly associated with the consumption of soft drinks and distilled alcoholic beverages and negatively associated with the consumption of raw food, coffee, and tea. It was also related to a high prevalence of current smokers and high energy intake. Despite the above, in the highest quartile of adherence, participants tended to have a clear increase in anti-inflammatory biomarkers, a hypolipidemic and hypotensive profile, as well as healthier anthropometrics. However, this pattern presents some elements of concern: lower HDL-c and higher insulin levels. The latter is in accordance with a higher consumption of soft drinks [42], and it could be explained by the effect of sweeteners on glucagon-like-peptide 1 (GLP-1), that increases insulin secretion [42,43,44]. Regarding HDL-c levels, a study of 7343 participants from the United States had similar results [45]. It should be noted that, although regression model adjustment included several confounding factors (such as age, sex, energy consumption, physical activity, and morbidity), differences in age among quartiles are important, and residual confounding cannot be ruled out. Overall, this pattern will lead to concerns regarding health outcomes if maintained over a long period of time.

Concerning the social business pattern, participants with higher adherence were characterized by the consumption of an unelaborated diet with highly salted products (foods cured with salt or smoke, cured cheese) as well as both fermented and distilled alcoholic beverages. They also had a high caloric intake probably due to the consumption of alcoholic drinks. At the same time, the consumption of alcoholic drinks could be responsible for some of the beneficial associations found, such as an increase in HDL-c. Generally, participants following this pattern had a low-quality diet and unhealthy lifestyles (they were more frequently current smokers, performed less physical activity, and had higher energy consumption). All these characteristics could explain their overall unhealthy cardiovascular profile. In particular, those adhering to this pattern showed a worse lipid profile (except for HDL-c) and renal function, an increase in diastolic blood pressure, as well as a detrimental association in anthropometrics. Concerning blood pressure, low dairy consumption could explain the increase in diastolic blood pressure [46]. Recently, the Progression of Early Subclinical Atherosclerosis (PESA) study carried out in Spain identified a dietary pattern called the social-business eating pattern [47]. This study included middle-aged participants with a high educational level, detrimental lifestyle habits, as well as a high consumption of alcoholic beverages and salt, sharing similar characteristics with our social business pattern. Considered as a whole, the social business pattern was the most detrimental for middle-aged participants.

Evidence from epidemiological studies on how cooking methods impact health is limited due to the difficulties with the collection of this information using population-based samples. The instruments most frequently used for this purpose (i.e., semi-quantitative food frequency questionnaires) do not allow distinguishing between food consumption and the cooking method used. At best, this procedure identifies some groups of foods that are consumed fried, such as fried potatoes or fried eggs. However, with the comprehensive dietary history applied to this study, detailed information on cooking/preservative methods was collected for each participant, being the main strength of the current study.

In this study, cooking and food preservation methods were comprehensively studied as a whole. When studying dietary patterns, we did not assess a single cooking method but a combination of all of them within a population. The main findings of our study are the following: The Spanish traditional pattern and the health-conscious pattern should be promoted. The youth-style pattern raises some concerns, and the social business pattern is clearly detrimental. However, more studies (including clinical trials) are necessary to allow us to evaluate the association between cooking patterns and their association with the incidence of cardio-metabolic disease and mortality. 

Therefore, this is the first time that cooking and food preservation patterns have being described, as well as their relationship with inflammatory and cardio-metabolic risk factors. In addition, the analyses were carried out with a large representative sample of the Spanish population, the data were collected using standardized protocols, and laboratory tests were centralized, reducing variability. 

This study also presented some limitations. The cross-sectional design did not allow us to establish causal inference. In addition, even though the dietary assessment was carried out by trained interviewers, some recall bias cannot be ruled out, which could lead to some degree of misclassification. Moreover, the analyses were adjusted for diseases diagnosed by a physician that were self-reported and might also lead to some misclassification in confounding variables. Finally, even if various confounders were considered, we cannot discard residual confounding. 

In conclusion, this study identified four cooking and food preservation patterns. These patterns showed associations with inflammatory and cardio-metabolic biomarkers. This analysis is the first step in the process of incorporating culinary methods and their impact on health when taking into account the complete diet.

## Figures and Tables

**Table 1:** Varimax-rotated factor-loading matrix obtained using factor analysis.

Cooking and Food Preservation Methods	Grams Mean (SD)	Spanish Traditional Pattern	Health-Conscious Pattern	Youth-Style Pattern	Social Business Pattern
Cooking methods					
Raw (not cooked)	356.5 (215.9)			−0.574	
Dairy products	276.9 (170.8)				−0.436
Boiling	271.4 (138.8)	0.398			
Roasting	197.0 (109.8)	0.426			
Pan-frying	66.8 (65.1)	−0.326			
Frying	58.2 (55.3)	0.370	−0.451		
Toasting	34.7 (44.0)		0.407		
Sautéing	33.5 (38.2)				
Stewing	25.1 (37.8)		−0.440		
Boiling and then sautéing	23.3 (26.9)	0.558			
Light frying (*sofrito*)	18.7 (21.9)	0.443			
Battering (bread crumbed)	9.1 (21.6)		−0.458		
Steaming	2.2 (10.5)		0.368		
Barbecuing	1.5 (10.5)				
Microwaving	0.8 (7.1)				
Preserving methods					
Curing cheeses	21.7 (33.1)				0.478
Curing (with salt or smoke)	17.8 (30.9)				0.487
Canning in oil	6.7 (11.2)	0.377	0.304		
Canning (not in oil)	5.2 (15.8)				
Brining	4.4 (10.3)	0.449	0.383		
Beverages					
Soft drinks	133.4 (252.1)			0.648	
Fermented alcoholic drinks	127.9 (243.2)				0.583
Coffee or tea	113.3 (127.1)			−0.400	
Distilled alcoholic drinks	4.6 (18.5)			0.464	0.336

Loading factors of <0.30 are not shown.

**Table 2:** Characteristics of the study participants by quartiles of adherence to the cooking and food preservation patterns. The ENRICA study 2008–2010.

	Spanish Traditional Pattern			Health-Conscious Pattern			Youth-Style Pattern			Social Business Pattern		
	Q1	Q4	*p*-Value Trend	Q1	Q4	*p*-Value Trend	Q1	Q4	*p*-Value Trend	Q1	Q4	*p*-Value Trend
	*n* = 2.315	*n* = 2.522		*n* = 2.516	*n* = 2.381		*n* = 2.464	*n* = 2.463		*n* = 2.452	*n* = 2.501	
Men, *n*, %	1188 (51.3)	1258 (49.9)	0.787	1273 (50.6)	1195 (50.2)	0.938	1257 (51.0)	1236 (50.2)	0.956	1217 (49.6)	1246 (49.8)	0.711
Age, y	47.9 (0.42)	47.1 (0.48)	0.389	47.2 (0.49)	46.0 (0.52)	0.188	55.2 (0.41)	36.0 (0.42)	<0.001	47.8 (0.51)	46.6 (0.52)	0.016
Education, *n*, %			0.114			<0.001			<0.001			<0.001
Primary	693 (29.9)	840 (33.3)		837 (33.3)	687 (28.9)		855 (34.7)	562 (22.8)		820 (33.4)	664 (26.5)	
Secondary	940 (40.6)	1022 (40.5)		1076 (42.7)	952 (40.0)		837 (34.0)	1340 (54.4)		976 (39.8)	1074 (42.9)	
University	682 (29.5)	660 (26.2)		603 (24.0)	742 (31.1)		772 (31.3)	561 (22.8)		656 (26.8)	763 (30.5)	
Smoking status, *n*, %			0.396			<0.001			<0.001			<0.001
Current smoker	653 (28.2)	660 (26.2)		769 (30.5)	612 (25.7)		499 (20.2)	898 (36.5)		524 (21.4)	850 (34.0)	
Former smoker	598 (25.8)	617 (24.5)		583 (23.2)	665 (27.9)		810 (32.9)	412 (16.7)		507 (20.7)	710 (28.4)	
Never smoker	1064 (46.0)	1245 (49.3)		1164 (46.3)	1104 (46.4)		1155 (46.9)	1152 (46.8)		1421 (57.9)	941 (37.6)	
Physical activity, MET-h/week	29.6 (21.5)	27.8 (23.0)	<0.001	26.3 (21.6)	31.0 (22.6)	<0.001	28.1 (20.1)	31.1 (24.8)	<0.001	28.8 (22.4)	28.2 (22.3)	<0.001
Time watching TV, h/week	14.0 (9.6)	13.7 (10.9)	<0.001	13.9 (10.5)	13.5 (10.0)	<0.001	14.2 (10.0)	13.2 (9.8)	<0.001	14.3 (10.3)	13.2 (9.9)	<0.001
Energy intake, kcal/day	1874.7 (549.3)	2600.1 (619.6)	<0.001	2372.2 (674.7)	2265.1 (602.3)	<0.001	2076.5 (573.7)	2457.0 (650.6)	<0.001	2023.7 (592.5)	2450.0 (624.0)	<0.001
Morbidity, *n*, %	943 (40.7)	1051 (41.7)	0.473	1070 (42.5)	920 (38.6)	0.138	1185 (48.1)	799 (32.4)	<0.001	1108 (45.2)	921 (36.8)	<0.001

MET: metabolic equivalents.

**Table 3:** Plasma and urine concentrations of inflammatory and cardio-metabolic biomarkers, blood pressure, and anthropometrics according to the quartiles of the Spanish traditional pattern.

	Spanish Traditional Pattern ^1^				
	Q1	Q2	Q3	Q4	*p*-Value for Trend
Biomarkers					
High-sensitivity C-reactive protein (Hs-CRP) (mg/L) ^2^	0.13 (1.42)	0.13 (1.41)	0.13 (1.44)	0.12 (1.46)	<0.001
Fibrinogen (g/L) ^2^	3.34 (1.09)	3.31 (1.09)	3.30 (1.09)	3.28 (1.09)	<0.001
Total cholesterol (mg/dL) ^2^	193.72 (1.07)	192.49 (1.07)	191.70 (1.07)	191.02 (1.07)	<0.001
LDL-cholesterol (mg/dL) ^2^	117.90 (1.08)	117.08 (1.08)	116.53 (1.09)	116.20 (1.09)	<0.001
HDL-cholesterol (mg/dL) ^2^	52.08 (1.12)	51.76 (1.13)	51.39 (1.13)	50.89 (1.14)	<0.001
Triglycerides (mg/dL) ^2^	95.70 (1.17)	94.70 (1.17)	94.81 (1.19)	95.06 (1.19)	0.425
Glucose (mg/dL) ^2^	92.14 (1.07)	91.49 (1.07)	91.31 (1.07)	91.13 (1.08)	<0.001
Insulin (μIU/mL) ^2^	7.46 (1.12)	7.45 (1.12)	7.51 (1.13)	7.58 (1.14)	<0.001
Homeostatic model assessment for insulin resistance (HOMA-IR) ^2^	1.70 (1.18)	1.68 (1.18)	1.70 (1.19)	1.71 (1.20)	0.319
Uric acid (mg/dL) ^2^	5.08 (1.17)	5.08 (1.18)	5.09 (1.19)	5.12 (1.20)	0.242
Urine albumin (mg/dL) ^2^	6.29 (1.21)	6.31 (1.21)	6.32 (1.22)	6.37 (1.23)	0.130
Urine albumin/creatinine ratio (mg/g creatinine) ^2^	5.34 (1.22)	5.26 (1.21)	5.26 (1.22)	5.24 (1.23)	0.034
Blood pressure					
Systolic blood pressure (mmHg)	129.52 (9.16)	128.59 (9.03)	128.25 (9.82)	128.24 (9.97)	0.002
Diastolic blood pressure (mmHg)	75.63 (3.11)	75.50 (3.09)	75.50 (3.32)	75.69 (3.38)	0.693
Anthropometrics					
Body mass index (kg/m^2^)	26.64 (1.95)	26.57 (1.91)	26.68 (2.09)	26.90 (2.15)	0.001
Waist circumference (cm)	89.91 (8.20)	89.96 (8.17)	90.52 (8.93)	91.52 (9.12)	<0.001
Waist-to-hip ratio	0.87 (0.06)	0.87 (0.06)	0.88 (0.07)	0.88 (0.07)	0.009

^1^ Values are adjusted for sex, age, educational level (primary, secondary, or university), smoking status (current, former, or never smoker), physical activity (MET-h/week), time spent watching TV (h/week), energy intake (kcal/day), and morbidity (yes, no). ^2^ Geometric means. LDL: Low density lipoprotein; HDL: high density lipoprotein.

**Table 4:** Plasma and urine concentrations of inflammatory and cardio-metabolic biomarkers, blood pressure, and anthropometrics according to the quartiles of the health-conscious pattern ^1^.

	Health-Conscious Pattern ^1^				
	Q1	Q2	Q3	Q4	*p*-Value for Trend
Biomarkers					
Hs-CRP (mg/L) ^2^	0.13 (1.45)	0.13 (1.43)	0.13 (1.42)	0.13 (1.42)	0.132
Fibrinogen (g/L) ^2^	3.29 (1.10)	3.32 (1.09)	3.32 (1.09)	3.31 (1.09)	0.048
Total cholesterol (mg/dL) ^2^	192.06 (1.07)	192.63 (1.07)	192.84 (1.07)	191.22 (1.07)	0.184
LDL-cholesterol (mg/dL) ^2^	117.00 (1.09)	117.29 (1.09)	117.28 (1.08)	116.00 (1.08)	0.018
HDL-cholesterol (mg/dL) ^2^	51.27 (1.14)	51.50 (1.13)	51.86 (1.12)	51.43 (1.13)	0.279
Triglycerides (mg/dL) ^2^	94.99 (1.19)	95.35 (1.18)	94.82 (1.18)	95.05 (1.18)	0.860
Glucose (mg/dL) ^2^	91.20 (1.08)	91.72 (1.07)	91.76 (1.07)	91.34 (1.07)	0.532
Insulin (U/mL) ^2^	7.49 (1.13)	7.49 (1.13)	7.47 (1.12)	7.54 (0.13)	0.263
HOMA-IR ^2^	1.69 (1.20)	1.70 (1.19)	1.70 (1.18)	1.70 (1.18)	0.344
Uric acid (mg/dL) ^2^	5.08 (1.19)	5.10 (1.18)	5.09 (1.18)	5.11 (1.18)	0.626
Urine albumin (mg/dL) ^2^	6.72 (1.24)	6.40 (1.22)	6.11 (1.20)	6.07 (1.21)	<0.001
Urine albumin/creatinine ratio (mg/g creatinine) ^2^	5.36 (1.23)	5.34 (1.22)	5.26 (1.21)	5.13 (1.21)	<0.001
Blood pressure					
Systolic blood pressure (mmHg)	129.00 (10.02)	129.02 (9.59)	128.60 (9.30)	127.87 (9.03)	0.001
Diastolic blood pressure (mmHg)	75.94 (3.40)	75.69 (3.26)	75.45 (3.13)	75.22 (3.08)	<0.001
Anthropometrics					
Body mass index (kg/m^2^)	26.73 (2.14)	26.68 (2.06)	26.63 (1.97)	26.75 (1.96)	0.969
Waist circumference (cm)	90.47 (9.15)	90.43 (8.73)	90.35 (8.41)	90.70 (8.31)	0.581
Waist-to-hip ratio	0.88 (0.07)	0.88 (0.07)	0.88 (0.06)	0.88 (0.06)	0.823

^1^ Values are adjusted for sex, age, educational level (primary, secondary, or university), smoking status (current, former, or never smoker), physical activity (MET-h/week), time spent watching TV (h/week), energy intake (kcal/day), and morbidity (yes, no).^2^ Geometric means. LDL: Low density lipoprotein; HDL: high density lipoprotein; Hs-CRP: High-sensitivity C-reactive protein; HOMA-IR: Homeostatic model assessment for insulin resistance.

**Table 5:** Plasma and urine concentrations of inflammatory and cardio-metabolic biomarkers, blood pressure, and anthropometrics according to the quartiles of the youth-style pattern ^1^.

	Youth-Style Pattern ^1^				
	Q1	Q2	Q3	Q4	*p*-Value for Trend
Biomarkers					
Hs-CRP (mg/L) ^2^	0.13 (1.43)	0.13 (1.45)	0.13 (1.47)	0.12 (1.42)	0.003
Fibrinogen (g/L) ^2^	3.38 (1.08)	3.35 (1.09)	3.32 (1.09)	3.19 (1.09)	<0.001
Total cholesterol (mg/dL) ^2^	200.39 (1.05)	195.46 (1.06)	191.24 (1.06)	182.16 (1.06)	<0.001
LDL-cholesterol (mg/dL) ^2^	123.49 (1.07)	119.48 (1.07)	116.09 (1.08)	109.02 (1.07)	<0.001
HDL-cholesterol (mg/dL) ^2^	53.07 (1.13)	52.16 (1.13)	51.07 (1.13)	49.82 (1.12)	<0.001
Triglycerides (mg/dL) ^2^	96.68 (1.19)	95.73 (1.18)	96.06 (1.19)	91.81 (1.17)	<0.001
Glucose (mg/dL) ^2^	94.17 (1.07)	92.63 (1.07)	91.49 (1.07)	87.84 (1.06)	<0.001
Insulin (μIU/mL) ^2^	7.22 (1.13)	7.41 (1.13)	7.67 (1.14)	7.71 (1.13)	<0.001
HOMA-IR ^2^	1.68 (1.19)	1.70 (1.19)	1.73 (1.20)	1.67 (1.18)	0.903
Uric acid (mg/dL) ^2^	5.20 (1.19)	5.13 (1.18)	5.10 (1.19)	4.97 (1.18)	<0.001
Urine albumin (mg/dL) ^2^	5.42 (1.19)	5.96 (1.19)	6.59 (1.20)	7.51 (1.18)	<0.001
Urine albumin/creatinine ratio (mg/g creatinine) ^2^	5.35 (1.21)	5.34 (1.22)	5.39 (1.24)	5.01 (1.22)	<0.001
Blood pressure					
Systolic blood pressure (mmHg)	131.78 (9.09)	129.93 (9.08)	128.70 (9.52)	124.11 (8.66)	<0.001
Diastolic blood pressure (mmHg)	77.08 (3.02)	76.11 (2.95)	75.42 (3.13)	73.71 (2.92)	<0.001
Anthropometrics					
Body mass index (kg/m^2^)	27.18 (1.99)	26.86 (2.00)	26.73 (2.08)	26.02 (1.88)	<0.001
Waist circumference (cm)	92.48 (8.59)	91.17 (8.39)	90.62 (8.77)	87.68 (8.14)	<0.001
Waist-to-hip ratio	0.89 (0.07)	0.88 (0.06)	0.88 (0.07)	0.85 (0.06)	<0.001

^1^ Values are adjusted for sex, age, educational level (primary, secondary, or university), smoking status (current, former, or never smoker), physical activity (MET-h/week), time spent watching TV (h/week), energy intake (kcal/day), and morbidity (yes, no).^2^ Geometric means. LDL: Low density lipoprotein; HDL: high density lipoprotein; Hs-CRP: High-sensitivity C-reactive protein; HOMA-IR: Homeostatic model assessment for insulin resistance.

**Table 6:** Plasma and urine concentrations of inflammatory and cardio-metabolic biomarkers, blood pressure, and anthropometrics according to the quartiles of the social business pattern ^1^**.**

	Social Business Pattern ^1^				
	Q1	Q2	Q3	Q4	*p*-Value for Trend
Biomarkers					
Hs-CRP (mg/L) ^2^	0.13 (1.48)	0.13 (1.45)	0.13 (1.40)	0.13 (1.38)	0.337
Fibrinogen (g/L) ^2^	3.37 (1.10)	3.34 (1.09)	3.28 (1.09)	3.25 (1.08)	<0.001
Total cholesterol (mg/dL) ^2^	188.31 (1.08)	191.10 (1.07)	193.31 (1.06)	196.03 (1.06)	<0.001
LDL-cholesterol (mg/dL) ^2^	114.51 (1.09)	116.29 (1.09)	117.59 (1.08)	119.21 (1.08)	<0.001
HDL-cholesterol (mg/dL) ^2^	50.65 (1.13)	51.19 (1.13)	51.78 (1.13)	52.43 (1.13)	<0.001
Triglycerides (mg/dL) ^2^	93.20 (1.19)	94.74 (1.18)	95.49 (1.18)	96.77 (1.18)	<0.001
Glucose (mg/dL) ^2^	91.59 (1.08)	91.73 (1.08)	91.40 (1.07)	91.31 (1.07)	0.128
Insulin (μIU/mL) ^2^	7.74 (1.12)	7.59 (1.12)	7.40 (1.12)	7.29 (1.12)	<0.001
HOMA-IR ^2^	1.75 (1.19)	1.72 (1.19)	1.67 (1.18)	1.65 (1.18)	<0.001
Uric acid (mg/dL) ^2^	4.97 (1.18)	5.07 (1.19)	5.15 (1.18)	5.20 (1.19)	<0.001
Urine albumin (mg/dL) ^2^	6.06 (1.22)	6.22 (1.22)	6.41 (1.21)	6.59 (1.21)	<0.001
Urine albumin/Creatinine ratio (mg/g creatinine) ^2^	5.27 (1.25)	5.29 (1.23)	5.24 (1.21)	5.28 (1.19)	0.994
Blood pressure					
Systolic blood pressure (mmHg)	128.89 (10.01)	128.94 (9.74)	128.46 (9.26)	128.26 (9.01)	0.038
Diastolic blood pressure (mmHg)	74.62 (3.31)	75.30 (3.26)	75.84 (3.14)	76.52 (3.13)	<0.001
Anthropometrics					
Body mass index (kg/m^2^)	26.51 (2.13)	26.67 (2.08)	26.71 (1.99)	26.90 (1.93)	<0.001
Waist circumference (cm)	90.00 (8.78)	90.45 (8.73)	90.52 (8.55)	90.97 (8.52)	0.004
Waist-to-hip ratio	0.87 (0.07)	0.88 (0.07)	0.88 (0.07)	0.88 (0.07)	0.037

^1^ Values are adjusted for sex, age, educational level (primary, secondary, or university), smoking status (current, former, or never smoker), physical activity (MET-h/week), time spent watching TV (h/week), energy intake (kcal/day), and morbidity (yes, no).^2^ Geometric means. LDL: Low density lipoprotein; HDL: high density lipoprotein; Hs-CRP: High-sensitivity C-reactive protein; HOMA-IR: Homeostatic model assessment for insulin resistance.

## Appendix A

**Table A1:** Cooking and food preservation methods.

Cooking Method	
Raw	Not cooked
Dairy products	Based on UHT milk (milk, yogurts, and fresh uncured cheese)
Boiling	To cook in water or liquids when the temperature is at boiling point
Roasting	To cook in an oven using hot air or radiation, which cooks the food evenly
Pan-frying	To cook food in a pan with the minimum amount of oil
Frying	To cook food in hot fats or oils when the food is totally or partially immersed
Toasting	Browning of the food using a grill and radiant heat
Sautéing	To cook food quickly with a few tablespoons of oil in a skillet over medium to high heat
Stewing	To boil slowly or simmering in a liquid for a long period of time at low heat
Boiling and then sautéing	Mixed method (boiling before sautéing)
Light frying (*sofreir*)	To cook slowly until golden over low to medium heat
Battering	To cover food with a liquid-type dough and then to fry
Steaming	To cook using vapor
Barbecuing	To cook on a rack over an open fire
Microwaving	To cook in an oven that uses microwaves
Preservation method	
Curing cheese	Aging cheese using methods like drying, salting, or smoking
Curing (with salt or smoke)	Drying food using salting or smoking
Canning in oil	To preserve food in a sealed can with oil
Canning (not in oil)	To preserve food in a sealed can with its natural juice, syrup, or vinegar
Brining	To preserve food in a concentrated solution of salted water
Beverages	
Soft drinks	Bottled carbonated sweetened drinks
Fermented alcoholic drinks	Wine and beer
Coffee or tea	Infusions
Distilled alcoholic drinks	Spirits with high alcohol content

**Table A2:** Relative changes in the adjusted estimators of extreme quartiles of inflammatory and cardio-metabolic biomarkers, blood pressure, and anthropometrics, according to the cooking and food preservation patterns.

	Spanish Traditional Pattern	Health-Conscious Pattern	Youth-Style Pattern	Social Business Pattern
Biomarkers				
Hs-CRP (mg/L)	−7.69%	NS	−7.69%	NS
Fibrinogen (g/L)	−1.79%	+0.60%	−5.62%	−3.56%
Total cholesterol (mg/dL)	−1.39%	NS	−9.09%	+3.93%
LDL-cholesterol (mg/dL)	−1.44%	−0.43%	−11.71%	+3.94%
HDL-cholesterol (mg/dL)	−2.28%	NS	−6.12%	+3.51%
Triglycerides (mg/dL)	NS	NS	−5.03%	+3.68%
Glucose (mg/dL)	−1.09%	NS	−6.72%	NS
Insulin (μIU/mL)	+1.58%	NS	+6.35%	−5.81%
HOMA−IR	NS	NS	NS	−5.71%
Uric acid (mg/dL)	NS	NS	−4.42%	+4.42%
Urine albumin (mg/dL)	NS	−9.60%	+27.82%	+8.04%
Urine albumin/creatinine ratio (mg/g creatinine)	−1.87%	−4.82%	−6.36%	NS
Blood pressure				
Systolic blood pressure (mmHg)	−0.98%	−0.87%	−5.82%	−0.50%
Diastolic blood pressure (mmHg)	NS	−0.95%	−4.37%	+2.48%
Anthropometrics				
Body mass index (kg/m^2^)	+0.97%	NS	−4.26%	+1.44%
Waist circumference (cm)	+1.75%	NS	−5.19%	+1.06%
Waist-to-hip ratio	+1.13%	NS	−4.49%	+1.13%

Figures in green indicate a beneficial relationship. Figures in red indicate a detrimental relationship. NS: Trend was not statistically significant. LDL: Low density lipoprotein; HDL: high density lipoprotein; Hs-CRP: High-sensitivity C-reactive protein; HOMA-IR: Homeostatic model assessment for insulin resistance.

## References

[1] Aranceta Bartrina J., Arija Val V., Maíz Aldalur E., Martínez de Victoria Muñoz E., Ortega Anta R.M., Pérez-Rodrigo C., Quiles Izquierdo J., Rodríguez Martín A., Román Viñas B., Grupo Colaborativo de la Sociedad Española de Nutrición Comunitaria (SENC) (2016). Dietary Guidelines for the Spanish population (SENC, diciembre 2016); the new graphic icon of healthy food. Nutr. Hosp..

[2] Barzegar F., Kamankesh M., Mohammadi A. (2019). Heterocyclic aromatic amines in cooked food: A review on formation, health risk-toxicology and their analytical techniques. Food Chem..

[3] Boskou G., Salta F.N., Chiou A., Troullidou E., Andrikopoulos N.K. (2006). Content oftrans,trans-2,4-decadienal in deep-fried and pan-fried potatoes. Eur. J. Lipid Sci. Technol..

[4] Guallar-Castillón P., Rodríguez-Artalejo F., Fornés N.S., Banegas J.R., Etxezarreta P.A., Ardanaz E., Barricarte A., Chirlaque M.-D., Iraeta M.D., Larrañaga N.L. (2007). Intake of fried foods is associated with obesity in the cohort of Spanish adults from the European Prospective Investigation into Cancer and Nutrition. Am. J. Clin. Nutr..

[5] Rey-García J., Guallar-Castillón P., Donat-Vargas C., Moreno-Iribas C., Barricarte A., Rodriguez-Barranco M., Colorado-Yohar S., Huerta J.-M., Chirlaque M.-D., Lasheras C. (2020). Fried-Food Consumption Does Not Increase the Risk of Stroke in the Spanish Cohort of the European Prospective Investigation into Cancer and Nutrition (EPIC) Study. J. Nutr..

[6] Guallar-Castillón P., Rodríguez-Artalejo F., Lopez-Garcia E., León-Muñoz L.M., Amiano P., Ardanaz E., Arriola L., Barricarte A., Buckland G., Chirlaque M.-D. (2012). Consumption of fried foods and risk of coronary heart disease: Spanish cohort of the European Prospective Investigation into Cancer and Nutrition study. BMJ.

[7] Sayon-Orea C., Bes-Rastrollo M., Basterra-Gortari F.J., Beunza J.J., Guallar-Castillon P., de la Fuente-Arrillaga C., Martinez-Gonzalez M.A. (2013). Consumption of fried foods and weight gain in a Mediterranean cohort: The SUN project. Nutr. Metab. Cardiovasc. Dis..

[8] Sayon-Orea C., Bes-Rastrollo M., Gea A., Zazpe I., Basterra-Gortari F.J., Martinez-Gonzalez M.A. (2014). Reported fried food consumption and the incidence of hypertension in a Mediterranean cohort: The SUN (Seguimiento Universidad de Navarra) project. Br. J. Nutr..

[9] Sayon-Orea C., Martinez-Gonzalez M.A., Gea A., Flores-Gomez E., Basterra-Gortari F.J., Bes-Rastrollo M. (2014). Consumption of fried foods and risk of metabolic syndrome: The SUN cohort study. Clin. Nutr..

[10] Muraki I., Rimm E.B., Willett W.C., Manson J.E., Hu F.B., Sun Q. (2016). Potato Consumption and Risk of Type 2 Diabetes: Results From Three Prospective Cohort Studies. Diabetes Care.

[11] Cahill L.E., Pan A., Chiuve S.E., Sun Q., Willett W.C., Hu F.B., Rimm E.B. (2014). Fried-food consumption and risk of type 2 diabetes and coronary artery disease: A prospective study in 2 cohorts of US women and men. Am. J. Clin. Nutr..

[12] Djoussé L., Petrone A.B., Gaziano J.M. (2015). Consumption of fried foods and risk of heart failure in the physicians’ health study. J. Am. Heart Assoc..

[13] Gadiraju T.V., Patel Y., Gaziano J.M., Djoussé L. (2015). Fried food consumption and cardiovascular health: A review of current evidence. Nutrients.

[14] Chinedum E., Sanni S., Theressa N., Ebere A. (2018). Effect of domestic cooking on the starch digestibility, predicted glycemic indices, polyphenol contents and alpha amylase inhibitory properties of beans (Phaseolis vulgaris) and breadfruit (Treculia africana). Int. J. Biol. Macromol..

[15] Mottram D.S., Wedzicha B.L., Dodson A.T. (2002). Food chemistry: Acrylamide is formed in the Maillard reaction. Nature.

[16] Sugimura T., Wakabayashi K., Nakagama H., Nagao M. (2004). Heterocyclic amines: Mutagens/carcinogens produced during cooking of meet and fish. Cancer Sci..

[17] Takachi R., Inoue M., Shimazu T., Sasazuki S., Ishihara J., Sawada N., Yamaji T., Iwasaki M., Iso H., Tsubono Y. (2010). Consumption of sodium and salted foods in relation to cancer and cardiovascular disease: The Japan Public Health Center-based Prospective Study. Am. J. Clin. Nutr..

[18] Hoffman R., Gerber M. (2015). Food Processing and the Mediterranean Diet. Nutrients.

[19] Koebnick C., Strassner C., Hoffmann I., Leitzmann C. (1999). Consequences of a Long-Term Raw Food Diet on Body Weight and Menstruation: Results of a Questionnaire Survey. Ann. Nutr. Metab..

[20] Koebnick C., Garcia A.L., Dagnelie P.C., Strassner C., Lindemans J., Katz N., Leitzmann C., Hoffmann I. (2005). Long-term consumption of a raw food diet is associated with favorable serum LDL cholesterol and triglycerides but also with elevated plasma homocysteine and low serum HDL cholesterol in humans. J. Nutr..

[21] Mozaffarian D., Gottdiener J.S., Siscovick D.S. (2006). Intake of tuna or other broiled or baked fish versus fried fish and cardiac structure, function, and hemodynamics. Am. J. Cardiol..

[22] Svelander C., Gabrielsson B.G., Almgren A., Gottfries J., Olsson J., Undeland I., Sandberg A.-S. (2015). Postprandial lipid and insulin responses among healthy, overweight men to mixed meals served with baked herring, pickled herring or baked, minced beef. Eur. J. Nutr..

[23] van Dusseldorp M., Smits P., Lenders J.W., Thien T., Katan M.B. (1991). Boiled coffee and blood pressure. A 14-week controlled trial. Hypertension.

[24] Hansen C.K., Klingenberg L., Larsen L.B., Lorenzen J.K., Sørensen K.V., Astrup A. (2017). The effect of UHT-processed dairy milk on cardio-metabolic risk factors. Eur. J. Clin. Nutr..

[25] Tian J., Chen J., Ye X., Chen S. (2016). Health benefits of the potato affected by domestic cooking: A review. Food Chem..

[26] Rodríguez-Artalejo F., Graciani A., Guallar-Castillón P., León-Muñoz L.M., Zuluaga M.C., López-García E., Gutiérrez-Fisac J.L., Taboada J.M., Aguilera M.T., Regidor E. (2011). Justificación y métodos del estudio sobre nutrición y riesgo cardiovascular en España (ENRICA). Rev. Española Cardiol..

[27] Guallar-Castillón P., Sagardui-Villamor J., Balboa-Castillo T., Sala-Vila A., Ariza Astolfi M.J., Sarrión Pelous M.D., León-Muñoz L.M., Graciani A., Laclaustra M., Benito C. (2014). Validity and reproducibility of a Spanish dietary history. PLoS ONE.

[28] Farrán A., Zamora R., Cervera P. (2004). Tablas de composición de alimentos del CESNID.

[29] Gutiérrez-Fisac J.L., Guallar-Castillón P., León-Muñoz L.M., Graciani A., Banegas J.R., Rodríguez-Artalejo F. (2012). Prevalence of general and abdominal obesity in the adult population of Spain, 2008–2010: The ENRICA study. Obes. Rev..

[30] Banegas J.R., Rodríguez-Artalejo F., Ruilope L.M., Graciani A., Luque M., de la Cruz-Troca J.J., García-Robles R., Tamargo J., Rey-Calero J. (2002). Hypertension magnitude and management in the elderly population of Spain. J. Hypertens..

[31] Friedewald W.T., Levy R.I., Fredrickson D.S. (1972). Estimation of the concentration of low-density lipoprotein cholesterol in plasma, without use of the preparative ultracentrifuge. Clin. Chem..

[32] Matthews D.R., Hosker J.P., Rudenski A.S., Naylor B.A., Treacher D.F., Turner R.C. (1985). Homeostasis model assessment: Insulin resistance and beta-cell function from fasting plasma glucose and insulin concentrations in man. Diabetologia.

[33] Pols M.A., Peeters P.H., Ocké M.C., Slimani N., Bueno-de-Mesquita H.B., Collette H.J. (1997). Estimation of reproducibility and relative validity of the questions included in the EPIC Physical Activity Questionnaire. Int. J. Epidemiol..

[34] Ainsworth B.E., Haskell W.L., Whitt M.C., Irwin M.L., Swartz A.M., Strath S.J., O’Brien W.L., Bassett D.R., Schmitz K.H., Emplaincourt P.O. (2000). Compendium of physical activities: An update of activity codes and MET intensities. Med. Sci. Sports Exerc..

[35] Harman H.H. (1976). Modern Factor Analysis.

[36] Slattery M.L., Boucher K.M., Caan B.J., Potter J.D., Ma K.-N. (1998). Eating Patterns and Risk of Colon Cancer. Am. J. Epidemiol..

[37] Ostan R., Lanzarini C., Pini E., Scurti M., Vianello D., Bertarelli C., Fabbri C., Izzi M., Palmas G., Biondi F. (2015). Inflammaging and Cancer: A Challenge for the Mediterranean Diet. Nutrients.

[38] Behera S.S., El Sheikha A.F., Hammami R., Kumar A. (2020). Traditionally fermented pickles: How the microbial diversity associated with their nutritional and health benefits?. J. Funct. Foods.

[39] Jonvik K.L., Nyakayiru J., Pinckaers P.J., Senden J.M., Van Loon L.J., Verdijk L.B. (2016). Nitrate-Rich Vegetables Increase Plasma Nitrate and Nitrite Concentrations and Lower Blood Pressure in Healthy Adults. J. Nutr. Nutr. Physiol. Metab. Nutr. Interact..

[40] Cao H., Ou J., Chen L., Zhang Y., Szkudelski T., Delmas D., Daglia M., Xiao J. (2019). Dietary polyphenols and type 2 diabetes: Human Study and Clinical Trial. Crit. Rev. Food Sci. Nutr..

[41] Sánchez-Muniz F.J. (2012). Dietary fibre and cardiovascular health. Nutr. Hosp..

[42] Sylvetsky A.C., Brown R.J., Blau J.E., Walter M., Rother K.I. (2016). Hormonal responses to non-nutritive sweeteners in water and diet soda. Nutr. Metab..

[43] Dotson C.D., Zhang L., Xu H., Shin Y.K., Vigues S., Ott S.H., Elson A.E.T., Choi H.J., Shaw H., Egan J.M. (2008). Bitter taste receptors influence glucose homeostasis. PLoS ONE.

[44] MacDonald P.E., El-kholy W., Riedel M.J., Salapatek A.M.F., Light P.E., Wheeler M.B. (2002). The multiple actions of GLP-1 on the process of glucose-stimulated insulin secretion. Proceedings of the Diabetes.

[45] Hert K.A., Fisk P.S., Rhee Y.S., Brunt A.R. (2014). Decreased consumption of sugar-sweetened beverages improved selected biomarkers of chronic disease risk among US adults: 1999 to 2010. Nutr. Res..

[46] Lana A., Banegas J.R., Guallar-Castillón P., Rodríguez-Artalejo F., Lopez-Garcia E. (2018). Association of Dairy Consumption and 24-Hour Blood Pressure in Older Adults with Hypertension. Am. J. Med..

[47] Peñalvo J.L., Fernández-Friera L., López-Melgar B., Uzhova I., Oliva B., Fernández-Alvira J.M., Laclaustra M., Pocock S., Mocoroa A., Mendiguren J.M. (2016). Association Between a Social-Business Eating Pattern and Early Asymptomatic Atherosclerosis. J. Am. Coll. Cardiol..

